# *In vitro* study of radiosensitivity in colorectal cancer cell lines associated with Lynch syndrome

**DOI:** 10.3389/fpubh.2024.1369201

**Published:** 2024-04-04

**Authors:** Mingzhu Sun, Jayne Moquet, Stephen Barnard, Hannah Mancey, David Burling, Rachel Baldwin-Cleland, Kevin Monahan, Andrew Latchford, David Lloyd, Simon Bouffler, Christophe Badie, Nicola A. Anyamene, Elizabeth Ainsbury

**Affiliations:** ^1^United Kingdom Health Security Agency, Department of Radiation Effects, Cytogenetics and Pathology Group, Radiation, Chemical and Environmental Hazards Directorate, Didcot, United Kingdom; ^2^Intestinal Imaging Centre, St Mark's Hospital, London North West University Healthcare National Health Service Trust, Harrow, United Kingdom; ^3^Lynch Syndrome Clinic, Centre for Familial Intestinal Cancer, St Mark's Hospital, London North West University Healthcare National Health Service Trust, Harrow, United Kingdom; ^4^East and North Hertfordshire National Health Service Trust, Mount Vernon Cancer Centre, Northwood, United Kingdom; ^5^Environmental Research Group Within the School of Public Health, Faculty of Medicine at Imperial College of Science, Technology and Medicine, London, United Kingdom

**Keywords:** Lynch syndrome, mismatch repair (MMR) deficiency, radiation effects, colorectal cancer cell lines, radiosensitivity

## Abstract

**Introduction:**

Lynch syndrome patients have an inherited predisposition to cancer due to a deficiency in DNA mismatch repair (MMR) genes which could lead to a higher risk of developing cancer if exposed to ionizing radiation. This pilot study aims to reveal the association between MMR deficiency and radiosensitivity at both a CT relevant low dose (20 mGy) and a therapeutic higher dose (2 Gy).

**Methods:**

Human colorectal cancer cell lines with (dMMR) or without MMR deficiency (pMMR) were analyzed before and after exposure to radiation using cellular and cytogenetic analyses i.e., clonogenic assay to determine cell reproductive death; sister chromatid exchange (SCE) assay to detect the exchange of DNA between sister chromatids; γH2AX assay to analyze DNA damage repair; and apoptosis analysis to compare cell death response. The advantages and limitations of these assays were assessed *in vitro*, and their applicability and feasibility investigated for their potential to be used for further studies using clinical samples.

**Results:**

Results from the clonogenic assay indicated that the pMMR cell line (HT29) was significantly more radio-resistant than the dMMR cell lines (HCT116, SW48, and LoVo) after 2 Gy X-irradiation. Both cell type and radiation dose had a significant effect on the yield of SCEs/chromosome. When the yield of SCEs/chromosome for the irradiated samples (2 Gy) was normalized against the controls, no significant difference was observed between the cell lines. For the γH2AX assay, 0, 20 mGy and 2 Gy were examined at post-exposure time points of 30 min (min), 4 and 24 h (h). Statistical analysis revealed that HT29 was only significantly more radio-resistant than the *MLH1*-deficient cells lines, but not the *MSH2*-deficient cell line. Apoptosis analysis (4 Gy) revealed that HT29 was significantly more radio-resistant than HCT116 albeit with very few apoptotic cells observed.

**Discussion:**

Overall, this study showed radio-resistance of the MMR proficient cell line in some assays, but not in the others. All methods used within this study have been validated; however, due to the limitations associated with cancer cell lines, the next step will be to use these assays in clinical samples in an effort to understand the biological and mechanistic effects of radiation in Lynch patients as well as the health implications.

## Introduction

Colorectal cancer (CRC) is the third most common cancer worldwide, with more than 1.9 million new cases contributing to 10.7% of all cancers in 2020 (www.wcrf.org). Lynch syndrome (LS) is the major cause of hereditary CRC as well as tumors at various other sites (e.g., endometrium, ovary, stomach, small bowel, urinary tract, biliary tract, brain, skin, pancreas, and prostate). LS is characterized by the heterozygous germline pathogenic variant in the coding sequence or regulatory domains of the mismatch repair (MMR) genes, most commonly *MLH1, MSH2, MSH6*, or *PMS2*. MMR proteins are associated with the detection and correction of DNA replication errors and a compromised MMR system can result in the mutator phenotype and the accumulation of somatic mutations can subsequently lead to carcinogenesis (1). LS is inherited in an autosomal dominant pattern. Carriers normally have one mutated allele of MMR gene and CRC develops when somatic mutation occurs to the wild-type allele. *MLH1* and *MSH2* mutations are typically associated with LS, whereas mutations in *MSH6* and *PMS2* are relatively rare (2). In the United Kingdom, an estimated 175,000 people have Lynch syndrome; however, fewer than 5% have been diagnosed due to a lack of awareness and systematic testing across the country (www.bowelcanceruk.org.uk). The improvement in diagnosis, treatment, and care for people with this condition is urgently required.

Due to the early onset age for LS (around mid-40's years of age), a 2-yearly colonoscopy surveillance is recommended for carriers of MMR pathogenic variants by the European Society of Gastrointestinal Endoscopy (3). Currently, CT colonoscopy is not recommended for large bowel surveillance regardless of its advantages over colonoscopy due to the unknown risk associated with ionizing radiation (IR). IR, such as X-rays used in CT scans and radiotherapy, can cause various types of DNA damage either directly by breaking the DNA strands or indirectly by the generation of reactive oxygen species and free radicals (4) even at low doses on diagnostic and surveillance levels. Therefore, the benefits of using medical radiation need to be balanced with the risks of harm following exposure. Currently, CT staging and surveillance of Lynch patients with CRC are routinely used world-wide due to the lack of published data or guidance for clinicians and radiologists regarding the relative risks of these patients in comparison to sporadic CRC patients. Additionally, although the benefits of CT colonography may significantly outweigh the radiation risks in the general population, CT associated malignancy could become significant with wider application of CT-based investigation (5). Importantly, neoadjuvant radiotherapy is routinely used for patients with advanced rectal tumors; however, the MMR status of these patients is not routinely considered in the pre-operative setting even though the information about the radiosensitivity of the tumor and its surrounding tissues is vital to support decision making (6).

Lynch syndrome as characterized by the deficiency in MMR genes is one of the few syndromes associated with a loss of biological functions that directly involve DNA damage recognition and repair (7). It is important for clinicians to be informed of the potential risks of radiation both for the surrounding tissues of the treatment area and for potential tumorigenesis following exposure because the presence of germline mutations may increase the risks of radiation toxicity and the development of secondary malignancies (8, 9).

To date, the radiosensitivity of Lynch patients at CT relevant low doses and therapeutic high doses of IR are largely unknown. As reported in a recent review, studies using LS associated primary cells or tumor cell lines exposed to both low and high doses of radiation showed contradictory results in radiosensitivity in terms of cell survival. Some of these cells were associated with higher mutation rates, which may have contributed to elevated cell death. Results from studies using animal models though showed increased radiation risk for dMMR mice potentially reducing the effectiveness of radiotherapy and increasing the risk of new cancer formation in the surrounding tissues (6).

The aim of this study is to investigate the radiosensitivity of LS-associated CRC cell lines, with or without MMR deficiency, using cellular and cytogenetic analytical approaches *in vitro*. This pilot study is part of a large project which aims to understand the radiation effects on Lynch syndrome patients with deficiency in MMR genes. MMR deficient colorectal cancer cell lines and an MMR proficient control were selected to test the applicability and limitations of the cellular and cytogenetic methods *in vitro*, which will be followed by the use of clinical samples. The clonogenic assay was used to assess cell reproductive death, sister chromatid exchange (SCE) analysis was used to examine homologous recombination repair, γH2AX assay was used to investigate DNA damage and repair and pan-caspase immunostaining was used to detect cell death response. Both a CT relevant low dose (20 mGy) and a radiotherapy related high dose (2 Gy) were used to study the effects of radiation on cell survival and DNA repair. 4 Gy was used for the apoptosis analysis due to the lack of positively stained cells at 2 Gy. Additionally, it was important to assess the applicability and feasibility of these methods for their potential to be used in clinical samples, and to evaluate the advantages and limitations of these assays during the study of mechanisms and pathways that may have a role in LS.

## Materials and methods

### Cell lines, chemicals, and reagents

MMR deficient and proficient cell lines (10, 11) were purchased from UK Health Security Agency (UKHSA) Culture Collections (Porton Down, UK). HCT116 (ECACC 91091005) is isolated from human colonic carcinoma with an epithelial-like morphology. SW48 (ECACC 89012702), LoVo (ECACC 87060101) and HT29 (ECACC 91072201) are adherent epithelial cells derived from human colon adenocarcinoma. HCT116 cells are *MLH1* deficient due to base substitution resulting in a termination signal at exon 9 codon 252. SW48 is also *MLH1* deficient resulting from promotor methylation. LoVo is *MSH2* deficient due to deletion of exon 3–8 (10). HT29 was used as an MMR proficient control. Further information about these cell lines can be found at www.culturecollections.org.uk.

Chemicals and reagents were purchased from Merck Life Science UK Ltd. (Gillingham, UK) unless otherwise specified. HCT116 and HT29 were cultured in McCoy's 5A medium. SW48 and LoVo cell lines were cultured in L-15 and Nutrient Mixture F-12 Ham medium, respectively. All these cell culturing media were supplemented with L-glutamine (2 mM), fetal bovine serum (10%) and antibiotics (100 units/mL penicillin and 100 μg/mL streptomycin). All cell lines were maintained in a 37°C incubator with 5% CO_2_ and 95% humidity except for SW48 which does not require CO_2_. All experiments were repeated three times except for the SCE, for which two repeats was considered sufficient based on power calculation.

### Irradiation

Irradiations were conducted at ambient temperature at a dose-rate of 0.5 Gy/min (250 kV, 13.0 mA) for 2 and 4 Gy, and 5 mGy/min (250 kV, 0.2 mA) for 20 mGy. The X-ray set (model CP160/1, Ago X-ray Ltd, Martock, UK) calibrated with reference to national standards with a half-value layer of Cu/Al filtration was used for all exposures. Each exposure was monitored using a calibrated UNIDOS E electrometer and “in-beam” monitor ionization chamber (all from PTW, Germany) (12). Due to the highly different detection sensitivity of each assay, 20 mGy was only used for γH2AX; 4 Gy was used for apoptosis analysis and 2 Gy was used for all assays in this study.

### Clonogenic assay

The clonogenic assay was performed in compliance with the protocols published by Brix et al. (13) and Franken et al. (14), which determines the proliferating fraction of cells capable of forming colonies with ≥50 cells in response to radiation. Briefly, single-cell suspension was generated by trypsinisation, washing in PBS and repeated trituration. Cells were then filtered through a cell strainer (40 μm, VWR International, Lutterworth, UK) to remove cell clusters. Trypan blue (0.4%, 1:1 dilution) assisted viable cell counting was conducted using a Neubauer Improved C*-*Chip™ hemocytometer (VWR International, Lutterworth, UK). Subsequently, cell suspension was diluted into desired seeding density and seeded into T25 flasks to protect cells from environmental contamination. Five hundred cells were seeded for non-irradiated control samples, and 1,500 (HT29) or 2,500 (HCT116, SW48, and LoVo) cells were seeded for irradiated samples with even distribution. Cells were irradiated at 2 Gy following adhesion (~4 h after seeding) and then kept for 10 (HT29, HCT116, and LoVo) or 15 (SW48) days under required conditions for the colonies to form. Cell culture medium was refreshed every 3 days. Colonies were eventually fixed with methanol:glacial acetic acid (3:1, v/v) and stained with crystal violet (0.5%) for counting. All four cell lines were treated in the same way under recommended culturing conditions, and the incubation time chosen for colony formation was based on preliminary testing that allowed at least six population doublings.

### Sister chromatid exchange (SCE) assay

The protocol for the SCE assay was based on the method published by Tumini and Aguilera (15). As each cell line has different cell cycle characteristics, initial experiments were carried out to determine the optimum time from seeding the cells to irradiation (T1) and from addition of 5-bromo-2′-deoxyuridine (BrdU, 10 μM final concentration) to harvest (T2), to ensure the cells in 2nd mitosis (M2 cells) would have been in G_1_ at irradiation (Table 1). 4 × 10^5^ cells were plated into a T25 flask from a confluent flask to ensure the cells were in exponential growth during BrdU incorporation. Depending on cell type, cells were irradiated with 2 Gy of X-rays at 5 h for fast growing cells (i.e., HCT116 and HT29), and 20 h for slow growing cells (i.e., LoVo and SW48), respectively. A zero-dose control was also included. Following irradiation, the medium was removed from all flasks and replaced with fresh medium containing BrdU. Colcemid (0.1 μg/mL) was added 2 h before termination of the cultures. After 23–52 h in culture, cells were harvested by trypsinisation and then treated with 0.075 M potassium chloride for 10 min at 37°C followed by three changes of methanol:glacial acetic acid fixative (3:1, v/v). Fixed cells were dropped onto clean microscope slides, air dried and stained by the fluorescence plus Giemsa (FPG) technique (16). Fifty M2 metaphase cells per sample were analyzed for SCEs. In addition, the relative numbers of cells in their 1st, 2nd, and 3rd division were assessed in 100 cells per sample to look for any changes in cell cycling speed caused by the radiation exposure by calculating the Nuclear Division Index (NDI) (4). For each cell type the SCE assay was performed on two separate occasions.

**Table 1:** The doubling time for different cell lines and the time points used in the setting up of sister chromatid exchange (SCE) analysis.

**Cell line**	**Doubling time (hours)**	**T1 time from setting up to irradiation (hours)**	**T2 time from addition of BrdU to fixation (hours)**
HT29	20^*^	5	25, 27, 29
HCT116	18^*^	5	23, 25, 27
SW48	35^*^	20	44, 52
LoVo	37^*^	20	30, 44

* The doubling time for HT29 is 20 h (https://www.cellosaurus.org/CVCL_0320); for HCT116 is 18 h (https://imanislife.com/collections/cell-lines/hct116-cells); for SW48 is 35 h (https://www.atcc.org/products/ccl-231); and for LoVo is 37 h (https://www.cellosaurus/CVCL_0399).

### γH2AX foci staining

Cells were processed using the protocol described by Rothkamm et al. (17). Briefly, cells were grown on sterile glass coverslips inside a 4-well plate at a density of 10^4^/mL. After 48 h, cells were irradiated with a desired dose (i.e., 20 mG or 2 Gy) at ~60–70% confluency alongside sham-irradiated controls. Cells were analyzed for γH2AX foci at 30 min, 4 and 24 h post-exposure. At these time points, cells were fixed for 5 min in phosphate buffered saline (PBS) with formaldehyde (4%), and permeabilized with Triton X-100 (1%) in PBS for 10 min. Subsequently, cells were blocked with bovine serum albumin (1%) for 30 min and immuno-stained for γH2AX (mouse anti-H2AX, 1:500 dilution, BioLegend^®^, London, UK) at room temperature for 1 h. Secondary antibody (goat anti-mouse conjugated with Alexa Fluor^®^ 555, 1:500 dilution, Fisher Scientific™, Loughborough, UK) and DAPI (1:500) were then added and incubated for 30 min at room temperature. Cells were eventually washed (3x5 min) in PBS to remove excess secondary antibodies before mounting with Vectashield Vibrance^®^ (Vector Laboratories, Kirtlington, UK). Slides were imaged using Nikon Ti-Eclipse fluorescent microscope equipped with NIS-Elements AR software (version 413.05). One thousand foci or 1,000 cells were scored for each condition, whichever came first based on power and sample size calculations.

### Apoptosis analysis

Caspases, a cascade of proteolytic enzymes, are the key effectors of apoptosis or programmed cell death. CHEMICON^®^ CaspaTag™ Pan-Caspase *in situ* assay kit (Scientific Laboratory Supplies Ltd., Hessle, UK) detects active caspases based on fluorochrome inhibitors of caspases and labels all the cells undergone apoptosis including those in the cell death process, thus showing an overall level of cell death over a period of time (18).

10^5^ cells (HT29 or HCT116) were seeded onto a sterile glass coverslip in each well of the 4-well plates and kept for 24 h in a 37°C incubator with 5% CO_2_ and 95% humidity. Cells were washed once in fresh medium to remove dead cells and irradiated at 4 Gy. Freshly prepared CaspaTag™ solution was added onto the cells 24 h post-exposure. Cells were incubated in CaspaTag™ solution for 80 min at 37°C, and then incubated with Hoechst for 10 min (0.5%) at 37°C to label the nuclei. Subsequently, the cells were washed three times in wash buffer, and fixed with formaldehyde (4%) at room temperature for 20 min. Cells were washed again for three times before the slides were mounted with Vectashield Vibrance^®^ antifade mounting medium for extended retention of staining.

Imaging was carried out using Nikon Ti-Eclipse microscope as described above. Fifty images were systematically taken and analyzed for each sample. All cells in focus were examined for the presence of positively stained apoptotic cells. The slow-growing cell lines (SW48 and LoVo; see Table 1 for doubling time) were not tested for apoptosis based on the findings from HT29 and HCT116, which showed very few positively stained cells even at 4 Gy.

### Statistics

For the clonogenic assay, one-way ANOVA and *post-hoc* analyses (Tukey's pairwise comparisons) were used to assess the difference in plating efficiency (PE) and surviving fraction (SF) for all four cell lines, and two-sample *t*-test was used to analyze differences in PE and SF within cell lines. For SCE assay, GLM ANOVA was used to analyze the effect of cell type and dose on the yield of SCEs/chromosome. Two-sample *t*-test was also used to compare the difference between the HT29 and HCT116 cell lines in apoptotic analysis. For the γH2AX assay, ANOVA and *post-hoc* analyses (Tukey's pairwise comparisons) were used to assess the difference in mean foci per cell between all four cell lines.

## Results

### Clonogenic assay

The 2 Gy dose was selected for clonogenic and the SCE assays in this pilot study because it is the average fraction dose for a standard radiotherapy session. Surviving fraction (SF) in response to radiation was calculated by normalization to the plating efficiency (PE) of the non-radiated control (14). SF was used as the indicator for radiosensitivity, the lower the value, the higher the sensitivity.


$$\begin{array}{c} PE=\frac{No.\;of\;colonies\;formed}{No.\;of\;cells\;seeded}\;x100\%\;\\ SF=\frac{No.\;of\;colonies\;formed\;after\;treatment}{No.\;of\;cells\;seeded\;x\;PE} \end{array}$$

**Figure 1:**
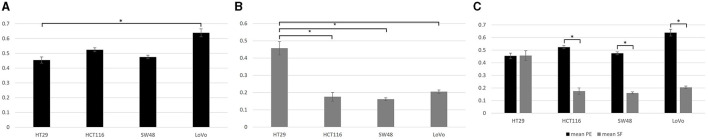
Data from clonogenic assay. **(A)** Plating efficiency (PE) for four colorectal cancer cell lines. Tukey pairwise analysis showed significant difference in mean PE value between only cell lines HT29 and LoVo (*P* < 0.04). **(B)** Surviving fraction (SF) for four colorectal cancer cell lines. SF for the MMR proficient cell line (HT29) was found to be significantly higher than those for the MMR deficient Cell lines (HCT116, SW48, and LoVo; all *p* < 0.0004). **(C)** The comparison between PE and SF showed that all MMR deficient cell lines had reduced capability to form colonies after irradiation (all *p* < 0.05); whereas the HT29 cell line showed no significant difference in the colony formation capability before and after irradiation at 2 Gy (*p* = 0.95). ^*^Indicates statistical significance.

Tukey pairwise analysis showed significant difference in mean PE value between only cell lines HT29 and LoVo (*P* < 0.04) and no significant difference was found between all the other cell lines (all *p* > 0.05; Figure 1A). The mean SF value for the MMR proficient HT29 was significantly higher than those for the *MHL1* deficient HCT116 and SW48 cell lines as well as for the *MSH2* deficient LoVo cell line (all *p* < 0.0004) using the same statistical analysis. These results suggested that the MMR proficient HT29 cell line may be significantly more radio-resistant than all the MMR deficient cell lines. No statistically significant difference was found in the mean SF value between the MMR deficient cell lines (all *p* > 0.5; Figure 1B). *T*-test also showed that for HT29 there was no significant difference in the colony formation capability before and after irradiation at 2 Gy (*p* = 0.95), whereas for all the MMR deficient cell lines, there was significantly reduced capability in colony formation after irradiation (all *p* < 0.05; Figure 1C).

### SCE assay

**Figure 2:**
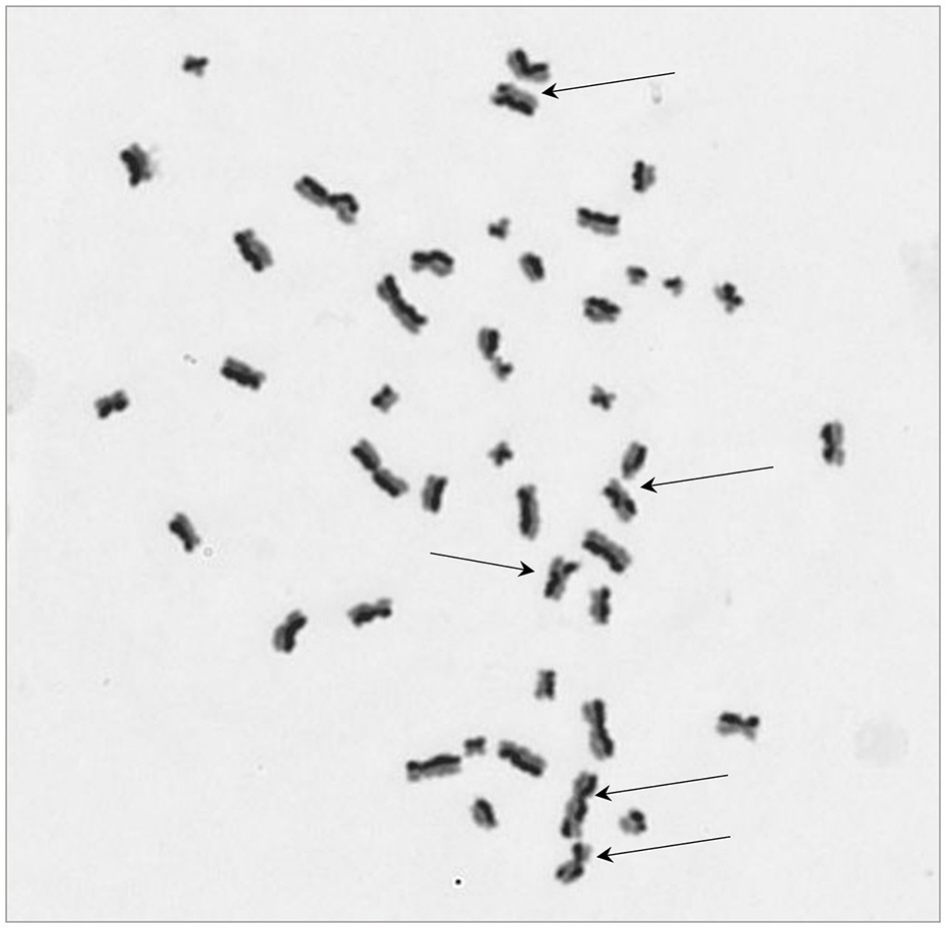
A representative image for sister chromatid exchange (SCE) showing differential integration of BrdU at 2nd cell division for a HCT116 cell. Arrows are pointing at chromosomes with SCE. The image was taken at 630x magnification.

The protocol worked well for all four cell lines and a representative image of a HCT116 cell (irradiated at 2 Gy) is shown in Figure 2 with arrows pointing to the SCEs. Table 2 shows the NDI derived from slides prepared for the SCE assay using the four cell types for two doses (0 and 2 Gy) and the repeat experiments. GLM ANOVA analysis indicated a significant difference between the two experiments and the dose (both *p* < 0.0001). As shown in Table 2, the NDI data for all the 2 Gy samples is less than the corresponding control and the NDI values for the second experiment tend to be lower than the first at both 0 and 2 Gy. Statistical analysis also revealed a difference between cell type (*p* = 0.007), and Tukey pairwise analysis indicated this was driven by the difference between the NDI values for HT29 and SW48 cells (*p* = 0.004).

**Table 2:** Nuclear Division Index (NDI) data for four CRC cell lines at two doses (0 and 2 Gy) and with a repeated experiment.

**Cell type**	**Time from addition of BrdU to fixation (h)**	**Dose (Gy)**	**Experiment repeat**	**M1**	**M2**	**M3+**	**Nuclear Division Index (NDI)**	**±SE**
HT29	25	0	1	62	38	0	1.38	0.12
			2	71	29	0	1.29	0.19
		2	1	85	15	0	1.15	0.25
			2	93	7	0	1.07	0.22
	27	0	1	40	60	0	1.6	0.10
			2	49	51	0	1.51	0.01
		2	1	64	36	0	1.36	0.13
			2	81	19	0	1.19	0.24
	29	0	1	19	81	0	1.81	0.24
			2	27	73	0	1.73	0.20
		2	1	41	59	0	1.59	0.09
			2	62	38	0	1.38	0.12
HCT116	23	0	1	55	45	0	1.45	0.05
			2	62	38	0	1.38	0.12
		2	1	82	18	0	1.18	0.25
			2	90	10	0	1.1	0.24
	25	0	1	23	77	0	1.77	0.23
			2	21	79	0	1.79	0.24
		2	1	57	43	0	1.43	0.07
			2	77	23	0	1.23	0.23
	27	0	1	10	90	0	1.9	0.24
			2	15	83	2	1.87	0.26
		2	1	31	69	0	1.69	0.18
			2	67	33	0	1.33	0.16
SW48	44	0	1	11	89	0	1.89	0.24
			2	8	92	0	1.92	0.23
		2	1	60	40	0	1.4	0.10
			2	83	17	0	1.17	0.25
	52	0	1	0	91	9	2.09	0.23
			2	6	94	0	1.94	0.21
		2	1	26	74	0	1.74	0.21
			2	44	56	0	1.56	0.06
LoVo	30	0	1	25	75	0	1.75	0.22
			2	60	40	0	1.4	0.10
		2	1	72	28	0	1.28	0.20
			2	90	10	0	1.1	0.24
	44	0	1	5	74	21	2.16	0.25
			2	18	82	0	1.82	0.25
		2	1	27	71	2	1.75	0.21
			2	42	58	0	1.58	0.08

M1, M2, and M3 are for 1st, 2nd, and 3rd divisions, respectively.

GLM ANOVA analysis indicated a significant difference between the two experiments and the dose (both p < 0.0001). It also revealed a significant difference between cell type (p = 0.007), which was driven by the difference between the NDI values for HT29 and SW48 cells (p = 0.004) as indicated by the Tukey pairwise analysis.

The results of the SCE assay are shown in Table 3. The cell types used in this study have a different modal number of chromosomes ranging from 46 to 68 (see Table 1). To allow comparison between the different cell types, the number of SCEs per chromosome was calculated. GLM ANOVA showed that cell type and dose had a significant effect on the yield of SCEs/chromosome (both *p* < 0.001). When the yield of SCEs/chromosome for the irradiated samples was normalized with their corresponding 0 Gy samples no significant difference was observed between the cell lines (*p* = 0.349).

**Table 3:** Sister chromatid exchange (SCE) data for four CRC cell lines at two doses (0 and 2 Gy) and with a repeated experiment including timeline, SCE yield and distribution.

**Cell type (modal- chromosome number)**	**Time from addition of BrdU to fixation (h)**	**Dose (Gy)**	**Experiment repeat**	**Total number of SCE in 50 cells**	**SCEdistribution**																			**SCE perchromosome**	**± SE**	**Yield of radiation induced SCE**	**± SE**
					**0**	**1**	**2**	**3**	**4**	**5**	**6**	**7**	**8**	**9**	**10**	**11**	**12**	**13**	**14**	**15**	**16**	**17**	**18**				
HT29 (68)	25	0	1	345	0	0	0	2	8	8	7	7	6	4	1	5	0	1	1					0.101	0.005		
			2	166	1	6	13	10	9	5	1	3	2											0.049	0.004		
		2	1	396	0	0	1	0	4	9	4	5	8	5	4	4	3	0	1	1	1			0.116	0.006	0.015	0.008
			2	225	0	1	7	11	10	7	8	1	1	2	1	1								0.066	0.004	0.017	0.006
	27	0	1	321	0	0	3	5	5	5	11	3	10	1	3	2	0	1	1					0.094	0.005		
			2	183	0	4	5	13	17	5	5	1												0.054	0.004		
		2	1	436	0	0	0	0	1	6	7	8	6	4	7	3	1	1	3	1	0	1	1	0.128	0.006	0.034	0.008
			2	265	0	3	4	5	9	7	5	6	6	3	1	1								0.078	0.005	0.024	0.006
	29	0	1	329	0	0	2	5	4	9	7	3	9	5	2	2	1	0	0	1				0.097	0.005		
			2	200	0	1	8	12	10	10	7	1	1											0.059	0.004		
		2	1	492	0	0	0	0	3	2	6	3	5	5	8	1	3	5	3	2	4			0.145	0.007	0.048	0.008
			2	264	0	0	4	5	9	10	11	6	3	0	0	1	1							0.078	0.005	0.019	0.006
HCT116 (46)	23	0	1	183	2	6	7	10	10	6	4	2	2	1										0.080	0.006		
			2	141	3	9	8	14	9	5	1	1												0.061	0.005		
		2	1	295	0	0	0	8	9	9	6	6	5	3	1	1	0	0	0	0	0	1		0.128	0.007	0.049	0.010
			2	180	0	4	12	11	9	5	7	1	0	1										0.078	0.006	0.017	0.008
	25	0	1	196	0	6	9	11	6	8	4	1	3	0	2									0.085	0.006		
			2	137	1	12	12	12	5	5	2	0	1											0.060	0.005		
		2	1	326	0	2	1	2	9	5	8	5	5	4	5	2	2							0.142	0.008	0.057	0.010
			2	163	0	3	13	16	11	3	2	1	1											0.071	0.006	0.011	0.008
	27	0	1	205	0	5	5	8	11	10	8	1	1	1										0.089	0.006		
			2	125	0	9	19	12	9	0	1													0.054	0.005		
		2	1	275	0	2	3	4	4	11	10	8	5	2	1									0.120	0.007	0.030	0.010
			2	174	0	5	9	14	10	6	3	3												0.076	0.006	0.021	0.008
SW48 (47)	44	0	1	135	0	11	17	8	7	5	1	1												0.057	0.005		
			2	107	4	16	13	6	8	3														0.046	0.004		
		2	1	208	1	2	9	5	14	7	7	3	0	1	1									0.089	0.006	0.031	0.008
			2	175	0	6	9	8	15	7	3	2												0.074	0.006	0.029	0.007
	52	0	1	141	2	10	9	10	14	3	2													0.060	0.005		
			2	125	0	12	17	12	5	2	1	1												0.053	0.005		
		2	1	217	0	1	7	11	9	11	3	5	2	1										0.092	0.006	0.032	0.008
			2	196	0	3	8	10	14	6	4	3	2											0.083	0.006	0.030	0.008
LoVo (49)	30	0	1	133	3	10	5	20	7	5														0.054	0.005		
			2	93	5	19	14	6	3	2	1													0.038	0.004		
		2	1	253	0	0	3	5	11	13	9	6	2	0	0	1								0.103	0.006	0.049	0.008
			2	190	0	2	9	10	14	8	6	0	1											0.078	0.006	0.040	0.007
	44	0	1	106	4	19	12	9	3	3														0.043	0.004		
			2	101	3	17	14	10	4	2														0.041	0.004		
		2	1	234	0	0	3	13	9	12	4	4	4	1										0.096	0.006	0.052	0.008
			2	154	5	18	10	7	7	1	2													0.063	0.005	0.022	0.007

GLM ANOVA showed that cell type and dose had a significant effect on the yield of SCEs/chromosome (both p < 0.001). However, when the yield of SCEs/chromosome for the irradiated samples was normalized with to their corresponding 0 Gy samples no significant difference was observed between the cell lines (p = 0.349).

### γH2AX

**Figure 3:**
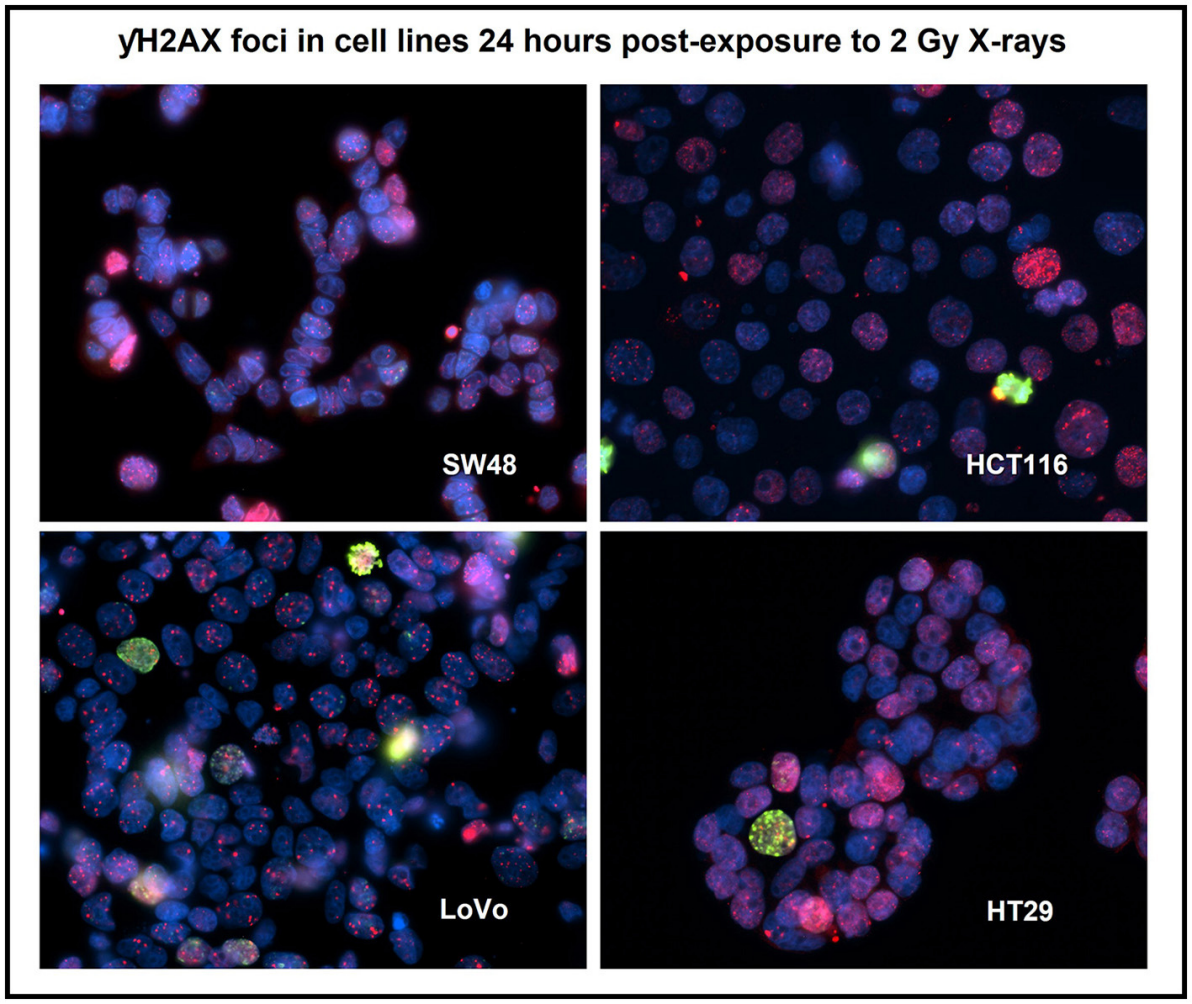
Immuno-staining for the four human colorectal cancer cell lines after 2 Gy X-irradiation using a DNA damage repair marker—γH2AX foci (red) and a cell proliferation marker—phosphorylated histone H3 (green). Nuclei were counter-stained with DAPI (blue). Staining was carried out 24 h post-exposure. Image was taken at 400x magnification. Cells with pan-nucleic staining were not scored.

Immunofluorescent staining allowed for the visualization of γH2AX foci before and after exposure. Mean foci per cell values were recorded for each exposure condition, time point and cell line as shown in Table 4. Control, 20 mGy and 2 Gy were included for time points 30 min, 4 and 24 h post-exposure. γH2AX foci were stained red and cell nuclei were counter-stained blue with DAPI. A cell proliferation marker—phosphorylated histone H3 was stained green using anti-phospho-H3 rabbit polyclonal antibody (Ser10) (06-570) conjugated with FITC (Figure 3). No significant difference was found for these cell proliferation markers for all the cell lines before and after irradiation, and the data are therefore not included in this study. Each variable of cell line, time post-exposure and dose were all statistically significant with regards to mean foci/cell values (all *p* ≤ 0.001).

**Table 4:** γH2AX assay data for four CRC cell lines at three doses (0, 20 mGy, 2 Gy) with three post-exposure time points.

**Cell line**	**Exposure dose**	**Post-exposure time points analyzed**		
		**30 min**	**4 h**	**24 h**
HT29	Control	2.76 ± 4.39	1.82 ± 1.31	1.91 ± 0.09
	20 mGy	4.39 ± 0.84		2.95 ± 0.16
	2 Gy		7.49 ± 0.35	3.42 ± 0.59
HCT116	Control	7.19 ± 1.81	5.06 ± 1.25	3.8 ± 1.15
	20 mGy	11.13 ± 6.46		4.43 ± 1.36
	2 Gy		10.33 ± 1.56	2.83 ± 0.31
SW48	Control	0.59 ± 0.23	0.82 ± 0.47	0.79 ± 0.41
	20 mGy	0.51 ± 0.15		1.02 ± 0.51
	2 Gy		2.57 ± 2.06	1.35 ± 0.78
LoVo	Control	2.06 ± 0.49	2.91 ± 0.81	1.75 ± 0.35
	20 mGy	1.92 ± 0.15		1.69 ± 0.17
	2 Gy		10.6 ± 1.19	2.98 ± 0.45

Mean foci/cell ± SE were recorded for each cell line, dose and time point.

With regards to mean foci/cell values, each variable of cell line, time post-exposure and dose were all statistically significant (all p ≤ 0.001).

**Figure 4:**
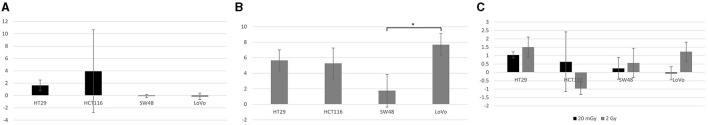
γH2AX foci assay data. **(A)** The normalized mean γH2AX foci per cell in four cell lines exposed to 20 mGy X-radiation and analyzed 30 min post-exposure. **(B)** The normalized mean γH2AX foci per cell in the same four cells lines but analyzed 4 h post-exposure to 2 Gy X-radiation. **(C)** The normalized mean γH2AX foci per cell in all four cell lines exposed to both 20 mGy and 2 Gy and analyzed 24 h post-exposure to X-radiation. All mean values normalized against control (not plotted here). Normalized standard error was plotted for all samples. No statistical difference was found between these cell lines except for the higher normalized mean γH2AX foci/cell for LoVo than that for SW48 after 2 Gy X-irradiation at the 4 h time point as shown in **(B)**. ^*^Indicates statistical significance.

Further Tukey pairwise analysis revealed no significant difference between LoVo and HT29 cell lines (*p* = 0.977); however, the responses of HCT116 and SW48 were significantly different from each other (*p* < 0.001) as well as LoVo and HT29 cell lines (*p* < 0.05). For time point, 4 and 24 h were significant from each other (*p* < 0.001), but not from 30 min. Finally, 2 Gy was significant from both control and 20 mGy (*p* ≤ 0.01), but there was no significant difference observed between control and 20 mGy (Figures 4A–C).

### Apoptosis analysis

**Figure 5:**
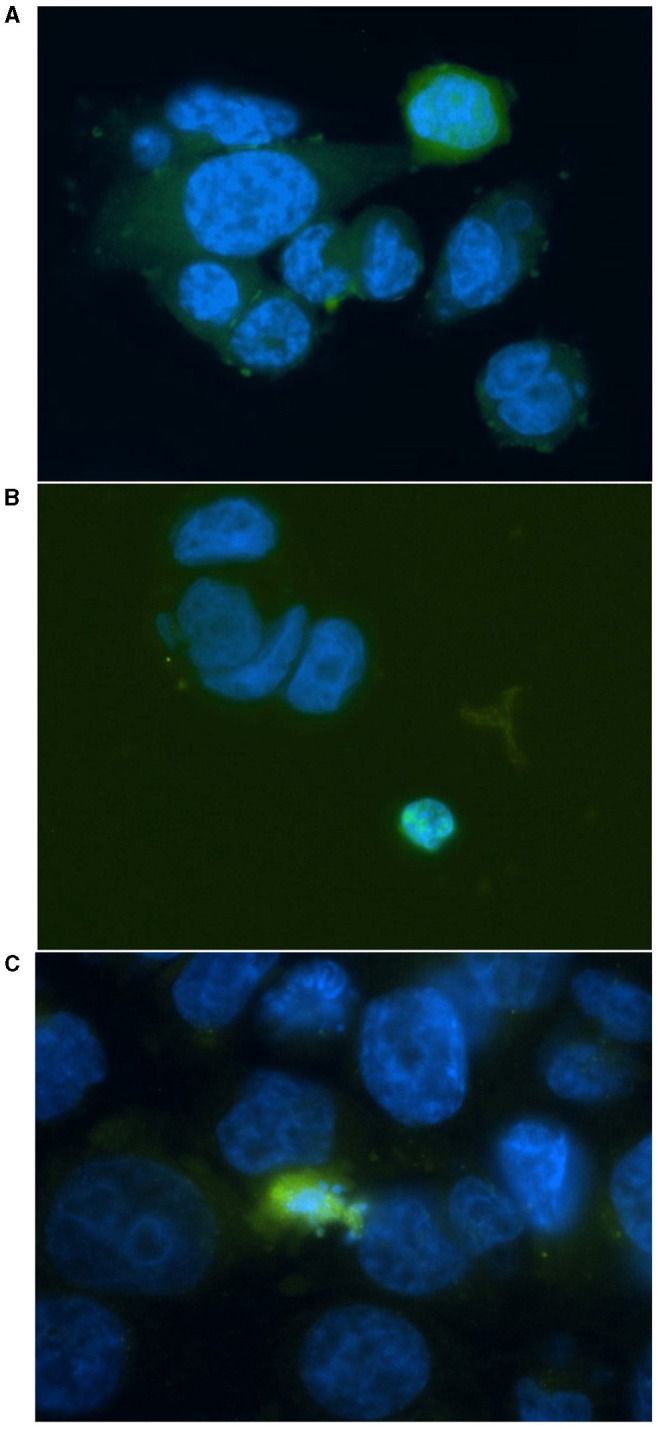
Representative images for positively stained HCT116 cells **(A, C)** and a HT29 cell **(B)** at different stages of apoptosis. Cells were irradiated at 4 Gy and stained with FITC-conjugated (green) CaspaTag™ Pan-Caspase *in situ* assay kit 24 h following irradiation. The nuclei were counter-stained with Hoechst (blue). Image was taken at 400x magnification.

**Figure 6:**
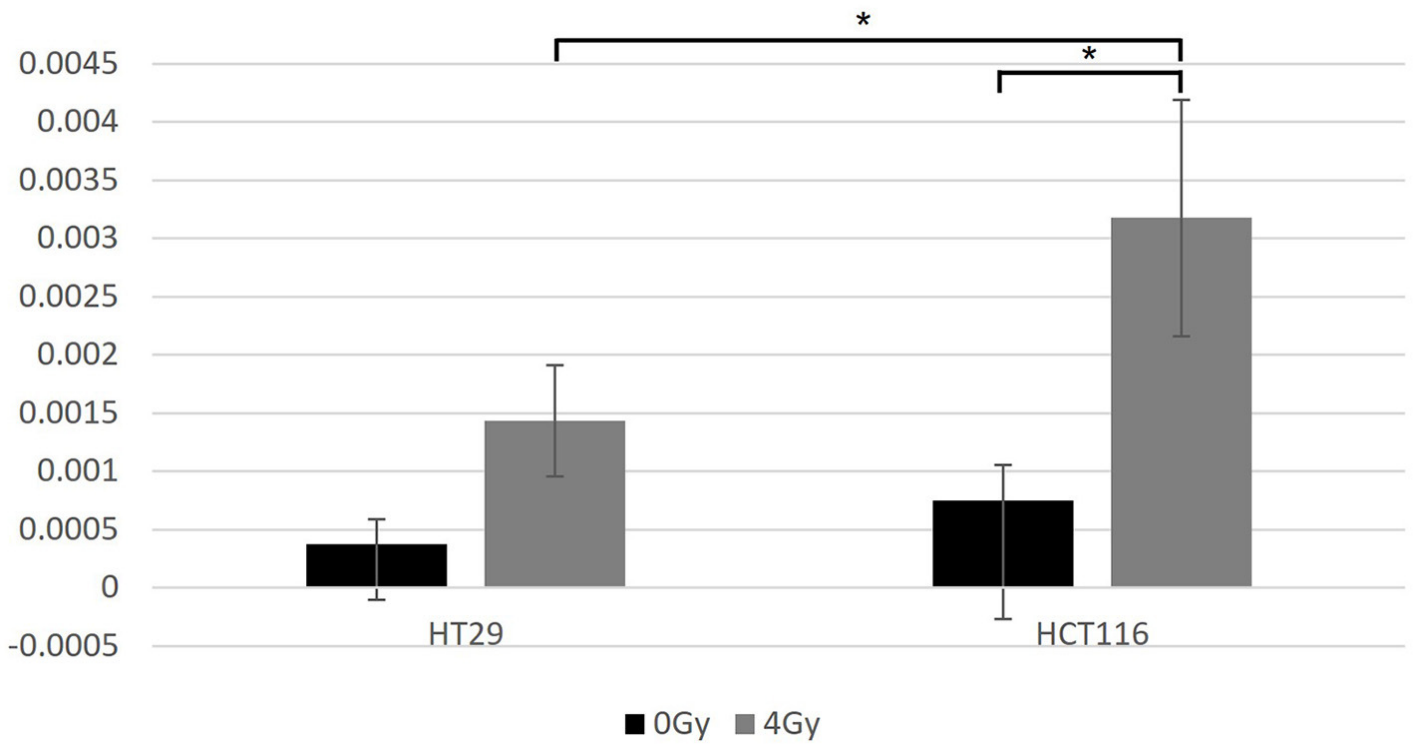
The data for the apoptosis analysis comparing the mean value of positively stained cells in each sample. Two-sample *t*-test showed significantly more positively stained cells for HCT116 than for HT29 only when exposed at 4 Gy (*p* = 0.015). Significantly more positively stained cells were found after irradiation for the HCT116 cell line (*p* < 0.05) when compared with non-irradiated control samples, but not for the HT29. ^*^Indicates statistical significance.

HT29 and HCT116 cell lines were examined for apoptosis at 0 and 4 Gy using CaspaTag™. A dose of 2 Gy was tested initially; however, due to the lack of positively stained cells, the exposure dose was increased to 4 Gy. Positively stained (green) HCT116 cells (A and C) and a HT29 cell (B) at different stages of apoptosis are shown in Figure 5, with nuclei counter-stained with Hoechst (blue). No apoptosis analysis was performed for the other two slow growing cell lines (i.e., SW48 and LoVo) based on the results for HT29 and HCT116 with very low level of positively stained cells. Figure 6 shows the data for the apoptosis analysis comparing the mean value of positively stained cells in each sample using the average results from three repeats. Two sample *t*-test revealed no statistically significant difference in the mean value for positively stained apoptotic cells in the HT29 cell line before and after irradiation. The mean value for the positively stained cells per sample in HCT116 was significantly higher at 4 Gy (*p* < 0.05) than at 0 Gy. The same statistical analysis showed significantly more positively stained cells for HCT116 than for HT29 when exposed to 4 Gy after subtracting the data from control samples (*p* = 0.015).

## Discussion and conclusion

Different types of tumor have distinctive patterns of DNA mutation and gene expression (19). The colorectal cancer cell lines selected within this study are not directly comparable in their radiosensitivity even though they were treated in the same ways wherever possible. These cell lines were selected and used mainly to test the feasibility and applicability of the cellular and cytogenetic analysis methods for the assessment of radiation-induced biological effects. The proficiency and deficiency in MMR genes may be associated with cell survival and DNA repair; nevertheless, the impact of radiation on the whole genome is what determines the fate of each cell line. The inherent defects in genome maintenance and repair are characteristic for cancer cell lines and therefore clinical samples with germline and/or somatic mutations in MMR genes, together with matched control samples, are required to further study the radiation effects on LS patients.

Both *MLH1* deficient and *MSH2* deficient cell lines were investigated for their radiosensitivity; however, the complex interaction mechanisms between MMR proteins and DNA before and after mismatch recognition are not well-understood. Briefly, MSH2 forms heterodimers with MSH6 or MSH3 depending on the nature and size of the mismatch; and the heterodimers subsequently bind to a complex containing MLH1/PMS2 heterodimer. The endonuclease activity of PMS2 is then initiated which leads to degradation of the mutated DNA and the restart of synthesis (20). MLH1 and MHS2 proteins have distinctively different features and roles in DNA mismatch repair; therefore, it is postulated that cells deficient in *MLH1* or *MSH2* respond differently to IR induced DNA damage. As such, the radiosensitivity of *MLH1* deficient cell lines (HCT116 and SW48) may be compared with the MMR proficient HT29 cell line, but not with the *MSH2* deficient cell line (LoVo). Similarly, radiosensitivity of LoVo cells may be compared with HT29, but not with HCT116 and SW48 cell lines. Additionally, HCT116 and SW48 are not comparable with each other as they have different underlying mechanism in *MLH1* deficiency.

### Clonogenic assay

At the cellular level, radiosensitivity corresponds to an excess of cellular death which is quantifiable by the clonogenicity of irradiated cells. Clonogenic cell survival is generally considered as the optimal method for the assessment and determination of radiosensitivity (21). The surviving fraction of cells at 2 Gy is one of the most reliable parameters for quantifying cellular radiosensitivity and is correlated with the *in vivo* radio-responsiveness (22). Our results show that the MMR proficient cell line (HT29) was more radio-resistant than the MMR deficient cell lines (HCT116, SW48, and LoVo) indicated by the significantly higher SF as well as the retained capability to form colonies after irradiation. Nevertheless, clonogenic assay cannot reveal the difference in the mechanisms of colony formation and this method cannot be used to detect CT relevant low doses. In addition, some cell lines show huge variability in terms of clonogenic survival due to genetic drift following long-term passage (23). Future work is intended using primary cells to further explore the radiosensitivity of tumors and the surrounding tissues without the complicated issues with mutations and DNA repair deficiencies associated with cancer cell lines such as those used within this present study.

### SCE

SCE assay allows the visual detection of DNA exchange between sister chromatids as the consequence of double-strand DNA (DSB) repair by homologous recombination (HR) during replication (24). SCEs are the result of DNA replication on a damaged template and can arise only when a DNA lesion is not removed before the cell enters S phase. MMR components function by suppressing HR repair between homologous sequences (25) and may subsequently lead to an additional level of genetic instability.

Our results in the lack of statistical difference for radiation induced SCEs may be explained by a previous discovery (26) which showed that microsatellite unstable colorectal cancer cell lines (often associated with MMR deficiency) are generally euploidy, less likely to undergo chromosomal gain, loss, or breakage than microsatellite stable cell lines. They also display significantly fewer cytogenetically evident alterations of chromosome structure. In contrast, microsatellite stable colorectal cancer cell lines typically display alterations not only of chromosome number but also of chromosome structure, such as chromosomal deletions, inversions, and translocations (26). Thus, MMR deficiency or proficiency may not have enough impact on the structural variation in the chromosomes of these cell lines.

Importantly, the results from SCE analysis may be explained by the fact that all four cell lines have no reported defect in *PMS2* and *MLH3* genes. It has been demonstrated that in human somatic cells, PMS2 and MLH3 promote DSB repair by HR and contribute as endonucleases independent of their functional activities in mismatch repair, whereas MLH1 and MSH2 are dispensable for HR (27). In another study (28), *MSH2* and nucleotide excision repair (NER) double deficient cell line, XP12ROB4, showed similar survival after exposure to γ-irradiation in comparison to the control cell line, XP12RO, with only NER deficiency. It was therefore postulated that MSH2 protein may not be involved in the process of IR-induced DNA damage and is unlikely to serve as a general sensor of DNA damage. Using these cell lines, similar level of spontaneous and UV-induced SCEs was also reported suggesting that MSH2 may not be involved in the recombination process (28).

Furthermore, radiation induced DNA DSBs trigger a cascade of cellular responses including checkpoint activation and cell cycle arrest at G_1_/S and G_2_/M, which allow the time for DNA repair (29). Unrepaired DNA may lead to senescence or apoptosis depending on the severity of the damage. There are two major DSB repair pathways: non-homologous end joining (NHEJ) and HR (30). NHEJ is an error-prone mechanism that directly ligates the break ends without using a template, whereas HR uses the undamaged sister chromatid as a template and enables error-free restoration. NHEJ operates throughout cell cycle and it is the predominant DSB repair pathway in mammalian cells (31). In contrast, HR is restricted to the S and G_2_ cell cycle phases (30). NHEJ associated repair only takes 1–2 h, whereas HR repair requires at least 8 h (30). It has been reported that in human cells DSBs induced by X-ray are predominately repaired by NHEJ (32, 33). Based on all these findings, SCE assay may not be a suitable method for the analysis of HR repair in LS associated CRC cell lines although primary cells isolated from Lynch patients may have a different cytogenetic profile.

### γH2AX foci

γH2AX is a phosphorylated variant of histone H2A and it is considered as one of the earliest markers of the DSB signaling (34). The γH2AX foci assay is a sensitive surrogate marker of radiation induced DSBs and a useful early biodosimetry tool from hours to about 3 days post-exposure (35). γH2AX foci form at the sites of DSBs and can be visualized within minutes of exposure; and importantly, induction of γH2AX foci is reported to be similar in both *in vitro* and *in vivo* settings (36). An intercomparison assessment for the feasibility of using γH2AX foci assay as a cellular marker for CT exposure showed that blood exposed at a dose point of 10 mGy could be distinguished from the non-irradiated control (*p* = 0.006) (37). Similarly, blood samples taken 5–30 min after chest-abdominal-pelvic CT or chest CT only (~5–20 mGy) showed significantly increased mean γH2AX foci yields when compared with the frequencies recorded for control blood samples taken prior to CT scan (36). Thus, the γH2AX foci assay was proposed as a sensitive and appropriate method for analyzing DNA damage and repair at diagnostic low-dose radiation levels.

Nevertheless, there are several limitations with the use of this method: (1) Established cells lines, especially those derived from tumors, can have higher background or spontaneous frequencies of γH2AX foci (38). (2) Cellular senescence, characterized by irreversible cell cycle arrest or proliferation arrest, can be induced in both normal and tumor tissue cells by sub-lethal dose of radiation (and other cellular stress factors, e.g., genotoxicity and oxidative stress); and cellular senescence is also associated with increased expression of histone γH2AX (39). Irradiated tumor cell lines are therefore expected to have a proportion of senescent cells that exhibit high levels of γH2AX foci. (3) γH2AX can also be a marker of telomere dysfunction and the phosphorylation of H2AX can occur independent of DNA damage (40). Based on these limitations, the results obtained from the present study cannot be interpreted for relative radio-sensitivity amongst the selected CRC cell lines. Nevertheless, this method can potentially be a valuable and feasible approach for the investigation of CT relevant low-dose exposures.

### Apoptosis analysis

Timelines for apoptosis vary for different cells even with the same inducing agent; and individual cells within the same seeding population can undergo apoptotic events at different times, therefore, time points were selected based on the general time course for cultured cell lines (41). Pro- and active caspases present in various subcellular locations including mitochondrial and cytosolic fractions, nuclear fraction and the microsomal fraction (42). Therefore, only cells stained positive in both cytoplasm and nucleus were scored as apoptotic.

In the present study, the small number of positively stained apoptotic cells at 4 Gy may be explained by: (1) Due to underlying genomic instability, cancer cells exhibit hallmark “anti-apoptotic” features which include sustaining proliferative signaling, enabling replicative immortality, and evading growth suppression (19). (2) Cancer cells can be induced into cellular senescence by sub-lethal environmental stresses such as IR (39) and when immortalized cells are propagated in culture, the repeated cycles of cell division can lead to cellular senescence. Senescent cells also exhibit apoptosis resistance (43). (3) For adherent cells, dead and dying cells experience changes in membrane integrity, become more rounded in morphology, and detach from the growth surface (www.leica-microsystems.com). These cells can easily be washed off during the staining procedure. However, the low detection level of this method may be improved by using primary cells isolated from clinical samples.

MMR genes are essential for the correction of DNA replication errors, and they also appear to be necessary for the induction of G_2_ cell cycle checkpoint and apoptosis. Nevertheless, it has been reported that MMR deficiency occurs as an early step in the development of tumors in LS (44). It plays the role by acquiring a mutator phenotype that drives the accumulation of mutations in genes required for the control of cellular growth and subsequently tumorigenesis (45). Therefore, the deficiency in MMR genes may not have direct effect on cell growth rate and apoptosis within a short period of time, but rather other effector genes are involved downstream. Accordingly, the time points used for future study may need to be extended and longitudinal samples examined to enable the potential to detect the effects of radiation for clinical samples *ex vivo*.

Importantly, radiosensitivity, defined as the predisposition to radiation induced adverse tissue reactions observed after radiotherapy, is different from the predisposition to radiation induced cancers termed as radiosusceptibility. Radiosensitivity attributable to cell death is generally correlated with un-repaired DNA damage, whereas radiosusceptibility is attributable to cell transformation and genomic instability as a result of mis-repaired DNA damage that is related to proto-oncogene or cell cycle control (46). This present study has used cytogenetic and cellular analysis tools to determine the radiosensitivity of cells, nevertheless, the *in vivo*, long-term effect of radiation on cell transformation requires longitudinal sampling and clinical monitoring.

In conclusion, four cellular and cytogenetic methods were used to investigate the radiosensitivity in four CRC cell lines following *in vitro* exposures to IR in terms of reproductive death, DNA damage and apoptosis. These analytical techniques employed within this study have demonstrated that they will be useful for studying the radiosensitivity of primary cells isolated from LS patients with either germline and/or somatic mutation in the MMR genes.

## Data availability statement

The original contributions presented in the study are included in the article/supplementary material, further inquiries can be directed to the corresponding author.

## Ethics statement

Ethical approval was not required for the studies on humans in accordance with the local legislation and institutional requirements because only commercially available established cell lines were used.

## Author contributions

MS: Conceptualization, Data curation, Formal analysis, Investigation, Methodology, Project administration, Resources, Validation, Writing – original draft, Writing – review & editing. JM: Data curation, Formal analysis, Investigation, Methodology, Supervision, Validation, Writing – review & editing. SBa: Data curation, Formal analysis, Investigation, Methodology, Writing – review & editing. HM: Writing – review & editing, Data curation, Formal analysis. DB: Writing – review & editing, Conceptualization, Funding acquisition. RB-C: Writing – review & editing, Investigation. KM: Writing – review & editing. AL: Writing – review & editing. DL: Writing – review & editing. SBo: Writing – review & editing. CB: Writing – review & editing. NA: Writing – review & editing. EA: Conceptualization, Funding acquisition, Writing – review & editing.
